# Supplementary material for: CD105 Is Expressed in Ovarian Cancer Precursor Lesions and Is Required for Metastasis to the Ovary
Source: Cancers (Basel). 2019 Nov 2;11(11):1710. doi: 10.3390/cancers11111710 (PMC6896092; doi:10.3390/cancers11111710)
Supplement: Supplementary file 1 [file cancers-11-01710-s001.pdf]

Supplemental Table 1. Two-way ANOVA analysis of IV mouse model of metastases.

ANOVA table	Sum of Squares	Degree of Freedom	Mean Sum of Squares	F value (DFn, DFd)	P value
Row Factor	3.9	3	1.3	F (3, 60) = 13.31	P<0.0001
Column Factor	4.474	1	4.474	F (1, 60) = 45.8	P<0.0001
Interaction	1.547	3	0.5158	F (3, 60) = 5.28	P=0.0027

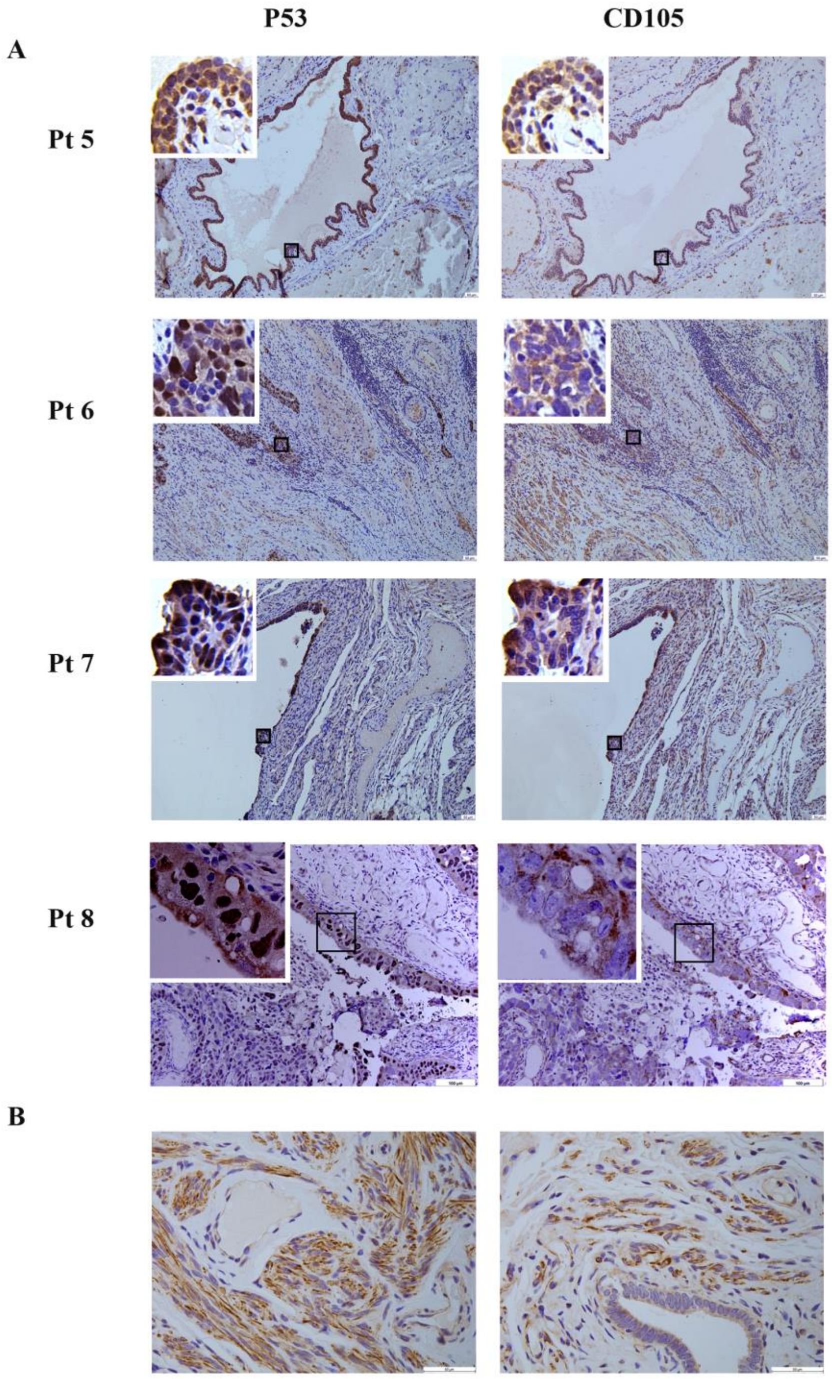

Supplemental Figure 1. **CD105 expression in Fallopian Tube STIC lesions and stromal cells in patient fallopian tube**. A. H&E eosin stain demonstrating histology of STIC lesions, (ii) p53 IHC confirming strong nuclear p53 expression in STIC lesions, and (iii) CD105 IHC demonstrating CD105 expression in STIC lesions. Boxed regions indicate regions of high power insets. Scale bars represent 50uM. highlight CD105, p53 expression in STIC with. B. Representative CD105 IHC demonstrating CD105 expression in stromal cells in patient fallopian tube.

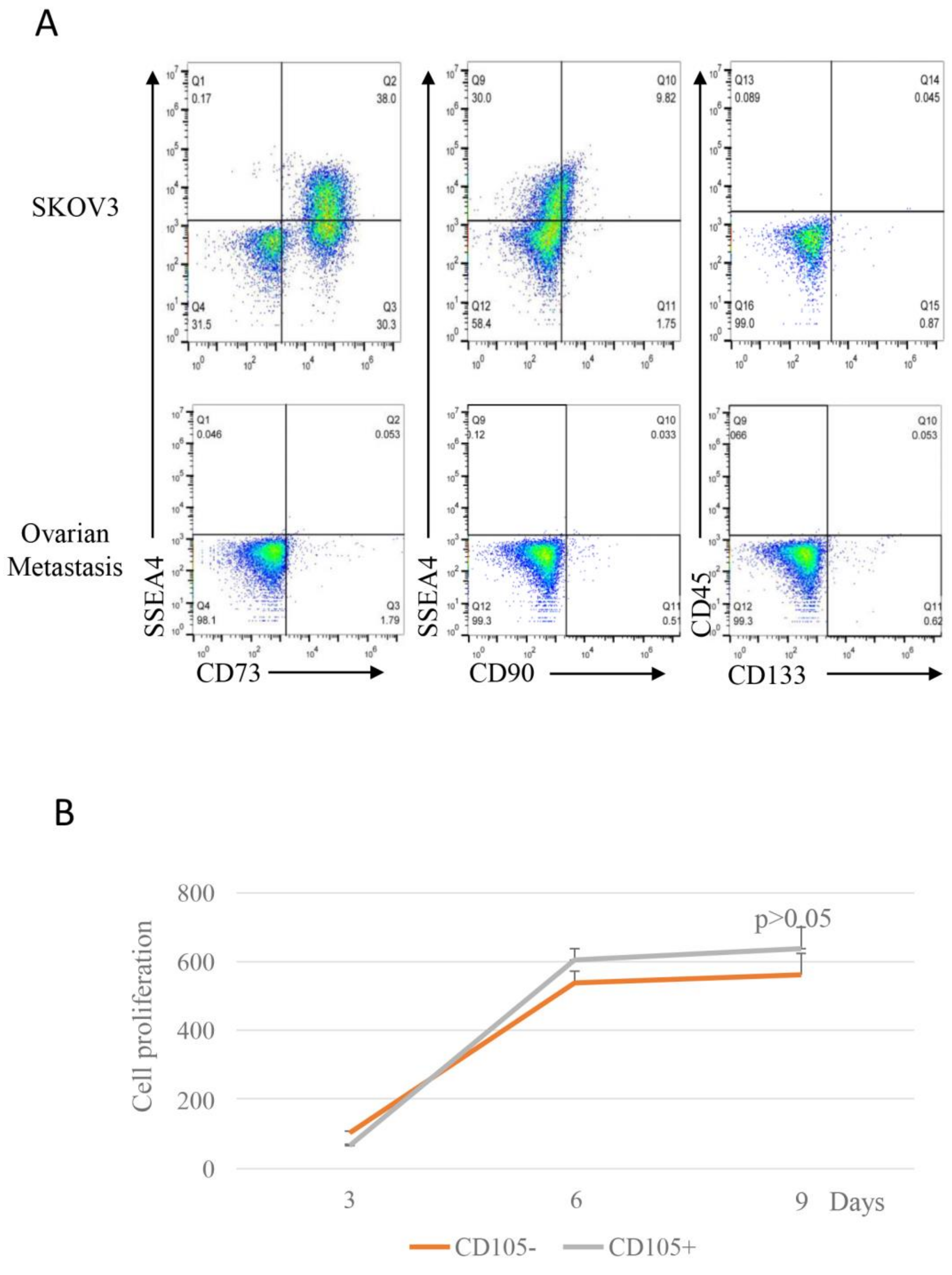

Supplemental Figure 2. Characterization of CD105 in SKOV3 cells. A. Flow cytometric analysis for the indicated cell markers in bulk SKOV3 cells in culture and cancer cells derived from an ovarian metastasis following IV-injected SKOV3. B. Cell proliferation rate of FACS-sorted CD105$^{(-)}$ and CD105$^{(+)}$ SKOV3 cells plated in tetrolates in 96-well plates. Cell proliferation were assessed by PrestoBlue cell viability reagent staining at times indicated. All data were normalized to control at Day 3 after plating, which was set as 100.

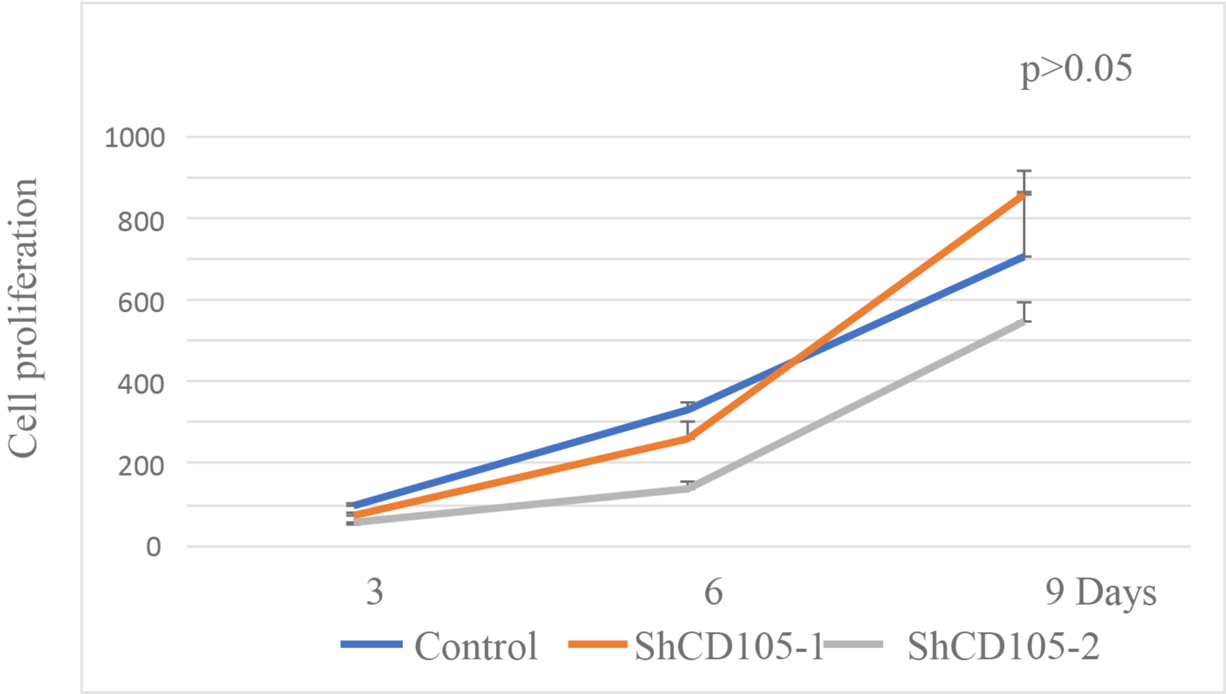

Supplemental Figure 3. Effect of CD105 knockdown on SKOV3 cell proliferation in vitro. Cell proliferation for CD105 knockdown cells (shCD105-1, shCD105-2) or scrambled shRNA (control) SKOV3cells. All data were normalized to control at Day 3 after plating, which was set as 100. Results are a summary of 2 independent assays with triplicate technical replicates.

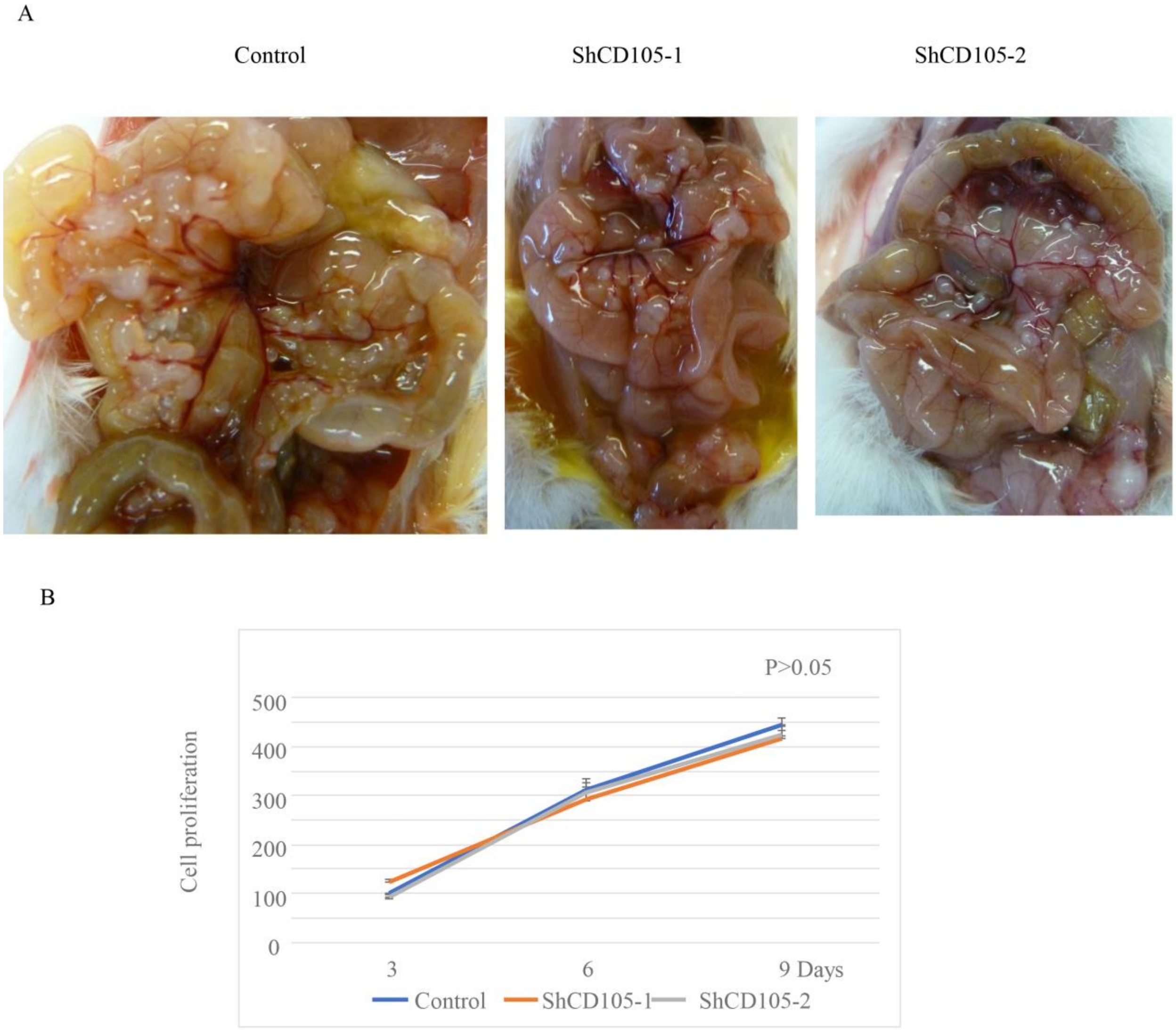

Supplemental Figure 4. Effect of CD105 knockdown on OVCAR3 metastatic growth *in vivo* and cell proliferation *in vitro*. A. Representative pictures of intra-abdominal tumor nodules in mice intraperitoneally injected with control and CD105 knockdown cells. B. Cell proliferation rate of CD105 knockdown cells and control OVCAR3 cells. Results are normalized to control cells 3 days after plating, which was set as 100.

Western Blot film for Figure 6F

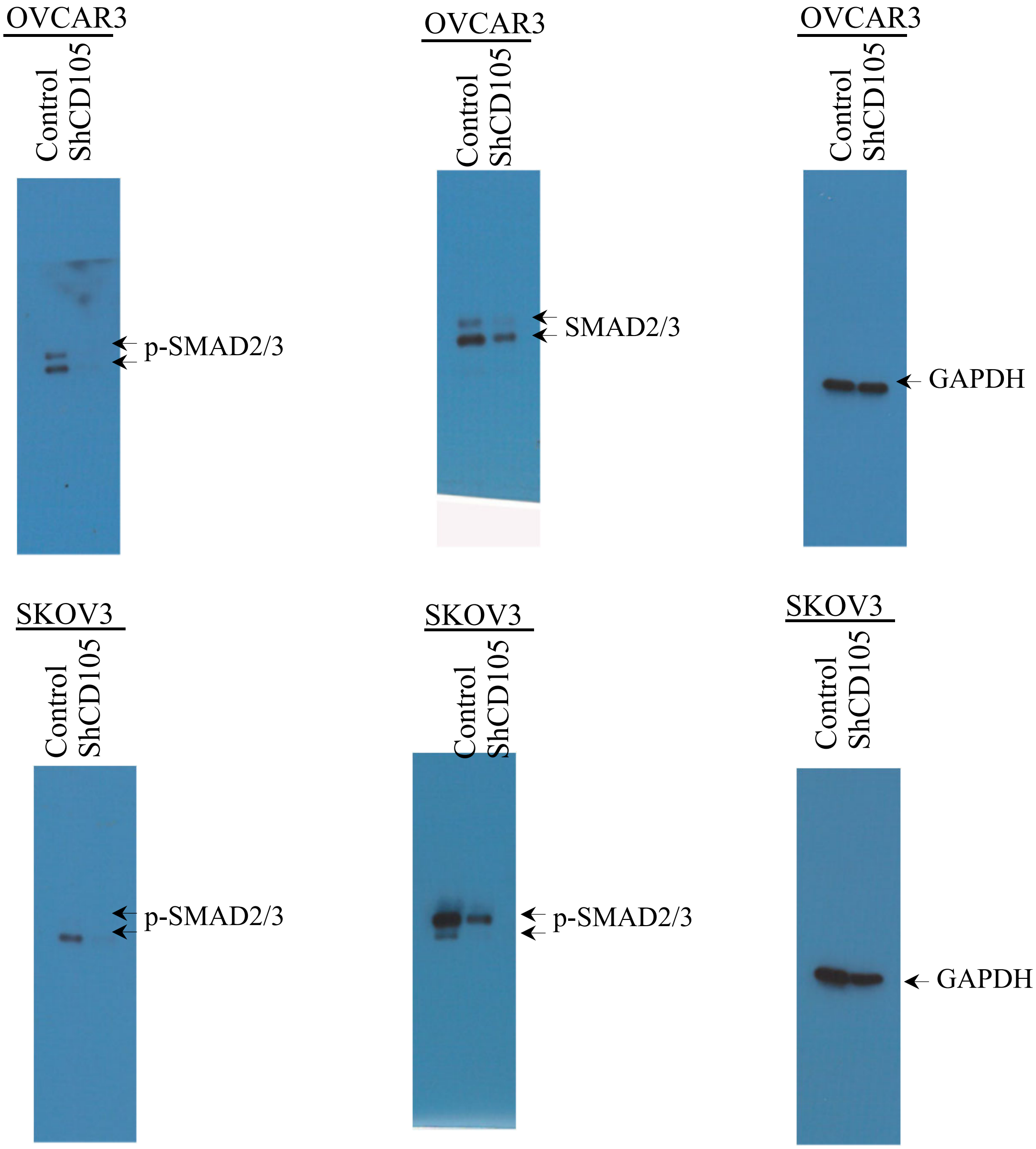

Densitometry Analysis of Western Blot in Figure 6F

		SKOV3			OVCAR3		
		Measure	Nomalized /GAPDH	Fold change	Measure	Nomalized /GAPDH	Fold change
Phospho	Control	17323.43	0.23	1.00	57443.06	1.08	1.00
SMAD2/3	ShCD105	2303.55	0.03	0.14	1756.38	0.04	0.03
Total	Control	68554.84	0.90	1.00	51003.61	0.96	1.00
SMAD2/3	ShCD105	11428.69	0.16	0.18	21290.61	0.43	0.44
GAPDH	Control	76406.186			52989.902		
	ShCD105	71794.337			49789.63		
